# Papillon-Lefèvre Syndrome: A Rare Case Report of Two Brothers and Review of the Literature

**DOI:** 10.5005/jp-journals-10005-1538

**Published:** 2018-08-01

**Authors:** Hytham N Fageeh

**Affiliations:** Diplomate and Vice Dean, Division of Periodontics, Department of Preventive Dental Sciences, College of Dentistry, Jazan University, Jazan, Kingdom of Saudi Arabia

**Keywords:** Palmar-plantar hyperkeratosis, Papillon-Lefèvre syndrome, Periodontitis.

## Abstract

Papillon-Lefèvre is an autosomal recessive syndrome that starts in early periods of childhood. Characteristic features include palmar plantar hyperkeratosis, aggressive periodontal disease, and a tendency for dry and chopped skin, thin and sparse hair. Patients show signs of premature tooth loss at the age of 2 to 4 years, which is then followed by the loss of permanent dentition during adolescence. The presence of both skin and oral lesions in this syndrome differentiates this unusual genodermatosis from other pathology of palmoplantar keratoderma (PPK). The etiopathogenesis of this syndrome is somewhat obscure; however, immunologic, genetic, and possible bacterial etiologies have been proposed. The dental practitioner is often the first to diagnose the disease, as there is a significant degree of periodontal breakdown that is involved at an early age. This report presents a clinical presentation of two brothers detected with Papillon-Lefèvre syndrome (PLS).

**How to cite this article:** Fageeh HN. Papillon-Lefèvre Syndrome: A Rare Case Report of Two Brothers and Review of the Literature. Int J Clin Pediatr Dent 2018;11(4):352-355.

## INTRODUCTION

Papillon-Lefèvre syndrome is an infrequent autosomal recessive disorder featured by destructive periodontitis beginning in childhood, diffuse, transgradient PPK, premature loss of permanent teeth, and frequent cutaneous and systemic pyogenic infections.^1,2^ Associated features also include susceptibility to bacterial infections, intra-cranial calcifications, and mental retardation.^3^

Variety of systemic conditions increases patient susceptibility to periodontal disease, leading to more rapid and aggressive attachment loss. The fundamental factors are chiefly related to changes in endocrine, immune, and connective tissue status.^4-6^ These modifications can be related to diverse pathologies and syndromes that produce periodontal disease either as a principal manifestation or by provoking a preexisting condition related to local factors.^1,2^ This article describes a clinical presentation of two brothers diagnosed with PLS.

## CASE REPORT

### Case 1

**Figs 1A to F:**
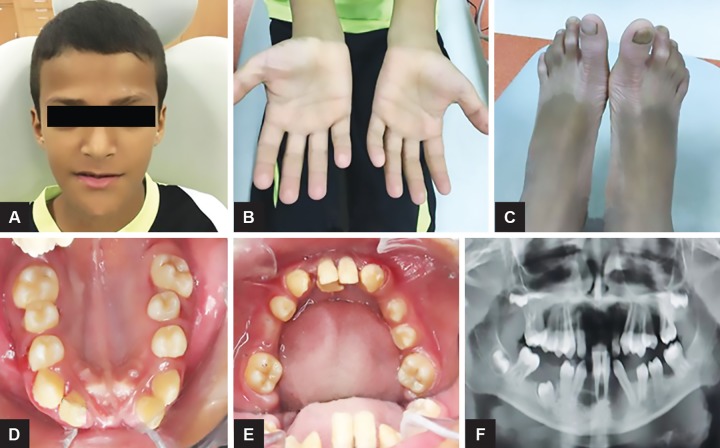
(A) A 12-year-old male patient; (B) palmar hyperkeratosis; (C) plantar hyperkeratosis; (D) intraoral clinical picture of the maxillary arch teeth; (E) intraoral clinical picture of the mandibular arch teeth; (F) orthopantomogram showing multiple loss of teeth and the alveolar bone loss around many present teeth

A 12-year-old male patient from Yemen (Fig. 1) reported to the clinic at the Preventive Dental Sciences Department at the College of Dentistry, Jazan University, with a complaint of mobile teeth for the last 6 months. According to the patient’s parents, his deciduous teeth erupted normally, but exfoliated at the age of 3. By the age of 10, the patient had multiple permanent teeth extracted due to mobility and now complains of mobility of the remaining permanent teeth. On physical examination, bilateral hyperkeratotic lesions on the palm and soles were observed; however, no signs of keratosis were present on the knees and elbows (Figs 1A to C). Family history showed that his parents were relatives and that his brother was also exhibiting similar complications.

Intraoral examination had shown presence of permanent maxillary right lateral incisor, canine, first premolar, first molar, permanent maxillary left canine, second premolar, and first molar. In mandible, permanent mandibular right central incisor, lateral incisor, canine, first premolar, first and second molars, left central incisor, canine, first and second premolar, and first molar were present. All other permanent teeth were missing (Figs 1D to F).

Of these teeth, maxillary right first molar, left second premolar, first molar, mandibular right canine, first premolar, and first molar were showing varying degrees of flaring and mobility. Severe gingival inflammation associated with thick plaque accumulation and deep periodontal pockets was present. Dermatological examination had shown the presence of symmetrical, well-demarcated keratotic plaques on the palms and soles. On radiographic examination, alveolar bone loss associated with all the affected teeth was noted. Also, third molar buds were present in their bony crypts, with normal crown development and no associated bony changes. On consideration of the clinical and radiological features, a diagnosis of PLS was made. The treatment plan included oral hygiene modification, nonsurgical periodontal therapy, extraction of all the remaining mobile teeth, and insertion of maxillary and mandibular dentures. Consideration of dental implants will be considered after the age of 18.

### Case 2

**Figs 2A to F:**
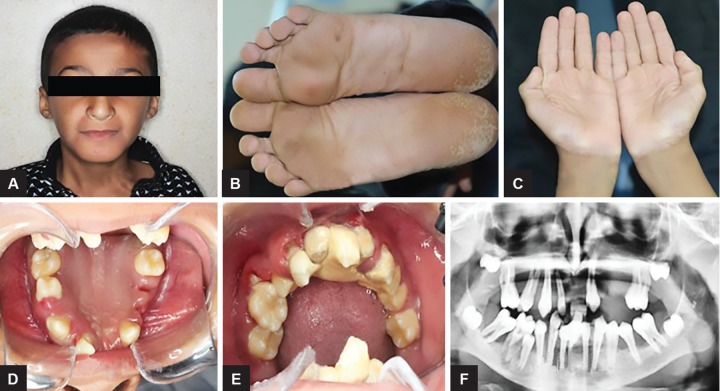
(A) An 11-year-old male patient; (B) plantar hyperkeratosis; (C) Palmar hyperkeratosis; (D) intraoral clinical picture of the maxillary arch teeth; (E) intraoral clinical picture of the mandibular arch teeth; (F) orthopantomogram showing multiple loss of teeth and the alveolar bone loss around many present teeth

The second case was the younger brother of the first patient who is an 11-year-old male (Fig. 2A). The patient reported a similar chief complaint of mobile teeth and past dental history from his parents also discovered that his deciduous teeth erupted normally and exfoliated at the same age of his brother. On physical examination, bilateral hyperkeratotic lesions on the palm and soles were observed; however, no signs of keratosis were present on the knees and elbows (Figs 2B and C).

Intraoral examination had shown the presence of permanent maxillary right canine, first and second premolar, first molar, permanent maxillary left lateral incisor, canine, first and second premolar, and first molar. In mandible, permanent mandibular right central incisor, canine, first premolar, first molar, left central incisor, canine, second premolar, and first molar were present. All other permanent teeth were missing. Of these remaining teeth, many were showing varying degrees of flaring mobility. Furthermore, severe gingival inflammation associated with thick plaque accumulation and deep periodontal pockets was present. Dermatological examination had shown the presence of symmetrical, well-demarcated keratotic plaques on the palms and soles. On radiographic examination, alveolar bone loss associated with all the affected teeth was noted. Also, third molar buds were present in their bony crypts except for the lower left third molar, with normal crown development and no associated bony changes. On consideration of the clinical and radiological features, a diagnosis of PLS was made. The treatment plan included oral hygiene modification, nonsurgical periodontal therapy, extraction of all the remaining mobile teeth, and insertion of maxillary and mandibular dentures (Figs 2D to F). On consideration of the clinical and radiological features, diagnosis of this patient was also made as PLS.

## DISCUSSION

Papillon-Lefèvre syndrome is an infrequent genodermal syndrome characterized initially by two French Physicians Papillon and Lefevre in 1924, in a brother and sister with palmoplantar hyperkeratosis accompanying with early onset periodontitis and premature loss of primary as well as permanent teeth. Gorlin et al in 1964 added a third section of dural calcification to the diagnosis of this syndrome.^7,8^ Almuneef et al further recognized pyogenic liver abscess to be a fairly associated complication.^3^

In the literature, several terminologies have been used for PLS: palmar-plantar hyperkeratosis with severe periodontal destruction involving both primary and permanent dentition, hyperkeratosis palmoplantaris with periodontitis, PPK with periodontitis, and keratosis palmoplantaris with periodontopathia.^9,10^

Papillon-Lefèvre syndrome has been categorized into three groups: diffuse, focal, and punctuated.^1^ The hyperkeratosis of the plantar surface often spreads to the edges of the soles and in some cases onto the skin overlying the Achilles tendon and the external malleoli. Other sites affected comprise the eyelids, labial commissures, cheeks, thighs, legs, and axillae. The hair is commonly normal, but the nails, in progressive cases, can show Assuring and transverse grooving.^11,12^

The dermatological features start appearing between the ages of 1 and 4 years and include lesions like palmo-plantar keratosis, varying from mild psoriasiform scaly skin to overt hyperkeratosis. The lesions are reported to be aggravated by cold.^3^ Both siblings showed similar signs of severe hyperkeratosis on both the palms and soles of the feet and intraoral features that included the presence of significant gingival inflammation, premature loss of teeth, mobility, bleeding on provocation, and severe periodontal destruction as those reported in case reports by Nickles et al^13^ and Vassilopoulou and Laskaris.^14^

Patients with PLS show periodontal destruction and severe gingival inflammation shortly after eruption of deciduous teeth, leading to the premature loss of the primary dentition causing the gingiva to recover its usual appearance. However, with the eruption of permanent teeth, an aggressive form of periodontal attachment loss is then re-triggered, often resulting in partial or complete loss of teeth that does not respond to conventional non-surgical periodontal therapy.^3^

Two important differential diagnoses of PLS are prepubertal periodontitis and Haim-Munk syndrome.

Haim-Munk syndrome is an allelic variation of PLS and the additional clinical features include arachnodactyly and acroosteolysis.^1,11^

Haneke used the following three criteria to classify a case as PLS^1,11^:

- Palmoplantar hyperkeratosis;
- Autosomal recessive inheritance; and
- Loss of primary and permanent teeth.

The prevalence of PLS is about 1 to 4 per million people. With both parents as recessive carriers, there is a 25% chance of generating offspring with this syndrome.^15^

The pathogenesis of PLS is still controversial. The skin lesions are supposed to be because of disorders in ectodermal and mesodermal constituents, but there is no cause to clarify the quick loss of all the deciduous as well as permanent teeth in the direction of their eruption.^7^

Two innovative aspects for the pathogenesis of PLS have been discovered. First, some patients suffering from PLS display a cellular immune fault with reduced chemotactic and phagocytic function of neutrophils and other granulocytes. Second, some pathogenic microorganisms like *Capnocytophaga gingivalis, Porphyromonas gingivalis, Actinobacillus actinomycetemcomitans, Peptostreptococcus micros, Fusobacterium nucleatum,* and spirochetes have been concerned as the causal agents for periodontal problems in PLS.^2,7,16^

Furthermore, a genetic tendency with greater incidence of occurrence in consanguineous offspring has been reported.^7^ Similarly, our patients had a history of parents of consanguineous marriage; however, this was not present in the case report by Rathi,^17^ who also reported a case of PLS in two Indian brothers.

Toomes et al^18^ demonstrated loss-of-function mutations disturbing both alleles of the lysosomal protease cathepsin C gene in PLS patients. The cathepsin C gene, which is situated on chromosome 11q14.1-q14.3 has endopeptidase activity and is expressed in epithelial regions normally exaggerated by PLS including palms, knees, soles, and keratinized oral gingiva. It is also stated at high levels in various immune cells including polymorphonuclear leukocytes, macrophages, and their precursors. Ryu et al^19^ trust that the severe periodontal destruction seen in PLS can be a result of loss of function mutation in the cathepsin C gene and following dysregulation of localized polymorphonuclear leukocytes in inflamed periodontal tissues.

Identification of PLS at an early age and a multidis-ciplinary approach can improve the prognosis of these patients. Skin lesions are commonly treated with emollients, salicylic acid, and urea. Oral retinoids including acitretin and isotretinoin have also been reported for the treatment of kertoderma.^20^ Effective treatment of periodontal disease includes prompt institution of antibiotics with nonsurgical therapy, the modification of the patient’s oral hygiene, extraction of primary teeth, and periodontal maintenance visit every 3 months. There is no consensus regarding the use of antibiotics; however, the prescription of antibiotics in combination with periodontal therapy has shown some benifit.^20-22^

## CONCLUSION

Papillon-Lefèvre syndrome can badly affect the psychological, social, and esthetic well-being of the patient at early age, as it is a devastating disease process associated with cutaneous involvement and partial or complete edentulism. The dental practitioner is usually the first to diagnose this syndrome due to the involvement of the periodontium. For the management of this condition, a multidisciplinary method is imperative, with psychological boost-up, importance on the dental and dermatological managing, and counseling of the affected individual.
